# Integrative practices of Canadian oncology health professionals

**DOI:** 10.3747/co.v15i0.283

**Published:** 2008-08

**Authors:** A.S.A. Brazier, L.G. Balneaves, D. Seely, J.E. Stephen, N. Suryaprakash, J.W. Taylor–Brown

**Keywords:** Oncology health professionals, integrative cancer care, qualitative research, complementary therapies

## Abstract

**Objective:**

Cancer patients are increasingly known to use complementary medicine (cam) during conventional treatment, but data are limited on how Canadian oncology health professionals attempt to assist patients with their use of cam in the context of conventional cancer care. As part of a larger qualitative study assessing the perceptions of Canadian oncology health professionals regarding integrated breast cancer care, we undertook an exploration of current integrative practices of oncology health professionals.

**Design:**

Using an interpretive description research design and a purposive sampling, we conducted a series of in-depth qualitative interviews with various oncology health professionals recruited from provincial cancer agencies, hospitals, integrative clinics, and private practice settings in four Canadian cities: Vancouver, Winnipeg, Montreal, and Halifax. A total of 16 oncology health professionals participated, including medical and radiation oncologists, nurses, and pharmacists.

**Results:**

Findings highlighted two main strategies used by oncology health professionals to create a more integrative approach for cancer patients:

- acting as an integrative care guide, and
- collaborating with other health professionals.

**Conclusions:**

Although few clear standards of practice or guidance material were in place within their organizational settings, health professionals discussed some integrative roles that they had adopted, depending on interest, knowledge, and skills, in supporting patients with cam decisions. Given that cancer patients report that they want to be able to confer with their conventional health professionals, particularly their oncologists, about their cam use, health professionals who elect to adopt integrative practices are likely offering patients much-welcomed support.

